# Exploring the Anti-Inflammatory Effects of *Aloe vera* Flower (AVF) and Its Active Ingredients in a Skin Inflammation Model Induced by Glyoxal-Derived Advanced Glycation End Products (GO-AGEs)

**DOI:** 10.3390/ph19010121

**Published:** 2026-01-09

**Authors:** Eun Yoo Lee, Seong-Min Hong, Sun Yeou Kim, Razia Sultana

**Affiliations:** 1 College of Pharmacy, Gachon University, 191 Hambakmoero, Yeonsu-gu, Incheon 21936, Republic of Korea; eunyoolee629@gmail.com; 2 Division of Food Science and Technology, Gyeongsang National University, Jinju 52828, Republic of Korea; hsm0206@gnu.ac.kr; 3 Gachon Institute of Pharmaceutical Science, Gachon University, 191 Hambakmoero, Yeonsu-gu, Incheon 21936, Republic of Korea; 4 Department of Life Science, University of Seoul, Seoul 02504, Republic of Korea; 5 Department of Pharmacy, Jagannath University, Dhaka 1100, Bangladesh

**Keywords:** *Aloe vera* flower, skin inflammaging, glyoxal-derived advanced glycation end products (GO-AGEs), anti-inflammatory, vitexin, isovitexin, Sirtuin1 (SIRT1)

## Abstract

**Objective**: Advanced glycation end-products (AGEs) contribute to oxidative stress and inflammation, leading to various disorders, including skin inflammation. Here, we investigated the anti-inflammatory effects of *Aloe vera* flower (AVF) extract and its active constituents, vitexin (V) and isovitexin (IV), in a glyoxal-derived AGE (GO-AGE)-induced skin inflammaging model. **Methods**: We evaluated the effects of AVF, V, and IV in epidermal keratinocytes (HaCaT cells) using enzyme-linked immunosorbent assay, Western blotting, quantitative real-time polymerase chain reaction, and in silico molecular docking. **Results**: Treatment of HaCaT cells with AVF, V, or IV significantly suppressed the secretion and expression of interleukins (IL-6 and IL-8) at both the mRNA and protein level, and reduced the expression of key inflammatory proteins, including kappa-light-chain-enhancer of activated B cells (NF-κB) and cyclooxygenase-2 (COX-2), and phosphorylation of mitogen-activated protein kinase (MAPK) pathway proteins. Notably, the inhibitory effects of V and IV on COX-2 expression were more comparable to or exceeded those of the positive control (Epigallocatechin gallate), even at a lower concentration. Conversely, the expression of sirtuin 1 (SIRT1) was upregulated by AVF, V, and IV, with IV showing 1.5-fold upregulation. Molecular docking analyses supported these findings, with IV displaying a particularly high binding affinity for COX-2 (−11.0 kcal/mol). **Conclusions**: These findings highlight the potential of AVF, V, and IV as novel therapeutic agents for managing skin inflammaging by modulating inflammatory pathways.

## 1. Introduction

Inflammation occurs as a response to injury and infection and plays essential roles in host defense and tissue repair [1]. However, excessive or prolonged inflammation can lead to chronic diseases such as psoriasis, diabetes, and cancer [2,3,4]. Inflammaging refers to a state of chronic, low-grade inflammation that occurs during aging, driven by endogenous signals that activate the innate immune system [4,5]. Advanced glycation end products (AGEs) contribute to oxidative stress and inflammation in disorders such as diabetes, arthritis, and skin inflammation [6,7,8]. Dicarbonyl compounds, including glyoxal (GO) and methylglyoxal (MGO), are precursors for AGEs formation [9,10]. GO-derived AGEs (GO-AGEs) are major contributors to skin inflammaging and associate conditions, including psoriasis and dermatitis [11,12]. Therefore, discovering potent anti-inflammatory agents from natural sources is a promising strategy for developing novel anti-inflammatory agents.

Epidermal keratinocytes form a protective barrier and act as immunocompetent cells by releasing various cytokines, including interleukin (IL)-6, IL-8, and IL-10 [13]. While classical stimuli such as ultraviolet-B (UVB) and lipopolysaccharide (LPS) are well-known inducers of skin inflammation, AGEs, especially GO-AGES, have emerged as significant pathological stimuli in various inflammatory skin disorders [6,11,13]. GO-AGEs have already been reported to induce inflammation, oxidative stress, etc., as reported through several studies [14,15,16]. Interestingly, we also demonstrated that GO-AGEs can induce inflammation in skin cells in our recent studies [16]. The inflammatory response in keratinocytes is mediated by the activation of key signaling cascades, particularly the mitogen-activated protein kinase (MAPK) and nuclear factor-kappa B (NF-κB) pathways. These pathways trigger the release of pro-inflammatory mediators, such as nitric oxide (NO), cyclooxygenase-2 (COX-2), and cytokines (e.g., IL-6 and IL-8), which drive skin aging and damage [17,18].

The majority of inflammation-associated disorders are linked to aging, making it critical to identify key molecular regulators of aging-associated inflammation. Sirtuins (SIRT 1–7), a class of nicotinamide adenine dinucleotide (NAD)-dependent histone deacetylases play important roles in this regulation, by modulating biological processes including aging, inflammation, and cancer, through the deacetylation of various histone and non-histone targets [19,20]. Several studies have shown the role of SIRT1 in mitigating inflammation, oxidative stress, and maintaining cellular homeostasis by regulating inflammation-associated signaling pathways [21,22].

*Aloe vera* (L.) Burm. (*Aloe barbadensis Mill*.) is widely recognized in pharmaceuticals and cosmetics because of its rich array of bioactive compounds, such as polysaccharides, anthraquinones, and flavonoids, which exhibit antioxidant and anti-inflammatory activities [23,24,25,26]. Recent studies have reported that the flowers of *A. vera* contain a higher concentration of polyphenols and show significantly greater antioxidant activity than its gel [27,28]. C-glycosyl flavonoids found in plants, especially isoorientin (IO), vitexin (V) and isovitexin (IV), possess promising pharmacological effects [29,30]. While our previous studies established that GO-AGEs induce skin inflammation, effective natural therapeutic agents targeting this specific pathway remain to be identified [16]. Furthermore, unlike the extensively studied *Aloe* gel, the *Aloe vera* flower (AVF) contains higher concentrations of bioactive polyphenols but has received limited attention. Therefore, in this study, we investigated the anti-inflammatory effects of AVF and their active constituents against GO-AGE-induced skin inflammaging in HaCaT keratinocytes, specifically focusing on the modulation of SIRT1 expression and downstream signaling pathways.

## 2. Results

### 2.1. AVF and Its Active Constituents Suppressed Pro-Inflammatory Cytokines Without Inducing Cytotoxicity

**Figure 1:**
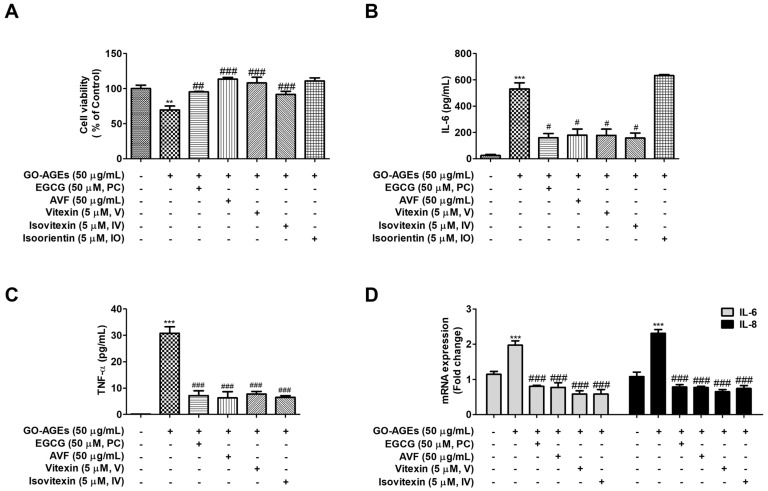
AVF and its active constituents suppress GO-AGE-induced inflammatory responses in HaCaT cells. (**A**) Cell viability assessed by MTT assay. (**B**) IL-6 and (**C**) TNF-α protein levels measured by ELISA. (**D**) Relative mRNA expression of IL-6 and IL-8 measured by qRT-PCR. Cells were treated with GO-AGEs (50 µg/mL) in the presence or absence of AVF (50 µg/mL), positive control (PC, EGCG 50 µM), vitexin (V, 5 µM), or isovitexin (IV, 5 µM). Data are expressed as the mean ± SEM of three independent experiments (*n* = 3). ** *p* < 0.01 and *** *p* < 0.001 vs. control; # *p* < 0.05, ## *p* < 0.01, and ### *p* < 0.001 vs. GO-AGE group.

The MTT assay was performed to evaluate the cytotoxicity of *Aloe vera* flower (AVF) and its active constituents. No cytotoxicity was observed with any of the cell treatments at the concentrations tested in HaCaT cells (Figure 1A). However, treatment with GO-AGEs significantly increased the production of the pro-inflammatory cytokines IL-6 and TNF-α (Figure 1B,C). To ensure consistent experimental conditions, cytokine measurements (ELISA) and gene expression analyses (qRT-PCR) were both conducted after a 24 h treatment period. This induction was reversed by subsequent treatment with AVF and its active constituents V and IV. However, unlike V and IV, IO failed to significantly inhibit the production of IL-6, a key pro-inflammatory cytokine (Figure 1B). Consequently, based on this lack of IL-6 inhibition in the primary screening, IO was excluded from further mechanistic investigations to focus on the most potent active constituents.

### 2.2. AVF and Its Active Constituents Inhibited Key Inflammatory Mediators

**Figure 2:**
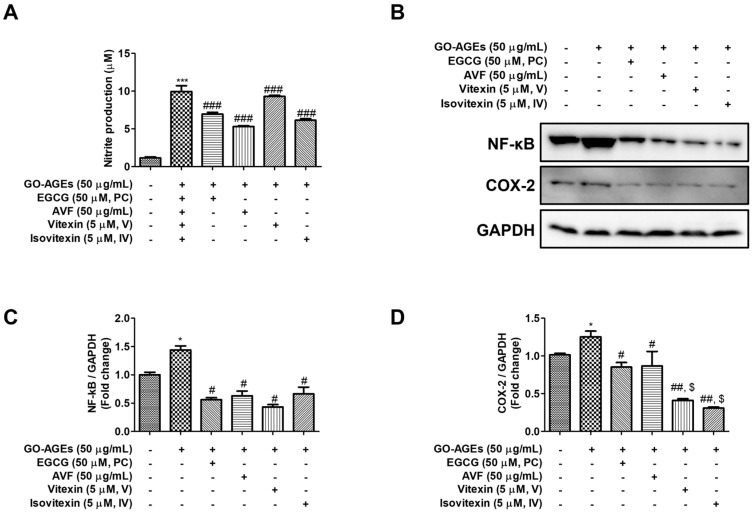
AVF and its active constituents inhibit GO-AGE-induced expression of inflammatory mediators in HaCaT cells. (**A**) Nitric oxide (NO) production measured by Griess assay. (**B**) Representative Western blot images for NF-κB and COX-2. (**C**,**D**) Densitometric quantification of NF-κB and COX-2 protein levels, normalized to GAPDH levels. Cells were treated with GO-AGEs (50 µg/mL) in the presence or absence of AVF (50 µg/mL), positive control (PC, EGCG 50 µM), vitexin (V, 5 µM), or isovitexin (IV, 5 µM). Data are expressed as the mean ± SEM of three independent experiments (*n* = 3). * *p* < 0.05 and *** *p* < 0.001 vs. control; # *p* < 0.05, ## *p* < 0.01, and ### *p* < 0.001 vs. GO-AGE group; $ *p* < 0.05 vs. PC group.

To investigate the underlying molecular mechanisms, we examined the production of key inflammatory mediators. Stimulation with GO-AGEs significantly increased the production of NO, a potent inflammatory signaling molecule, in HaCaT cells, which was reversed by AVF, V, and IV treatment (Figure 2A). Next, we assessed the expression of COX-2, a critical enzyme involved in inflammatory prostaglandin production. GO-AGEs significantly upregulated COX-2, which was effectively suppressed by the treatment of V and IV (Figure 2B,D). However, AVF was unable to inhibit the upregulatory effect of GO-AGEs on COX-2 expression. Western blot analysis revealed that AVF, V, and IV markedly attenuated GO-AGE-induced expression of NF-κB (Figure 2B,C). Notably, the inhibitory effects of V and IV on COX-2 expression were in a similar pattern of positive control, EGCG. These results suggest that AVF and its active constituents exert the anti-inflammatory effects by targeting the NF-κB signaling axis, which in turn suppresses the production of crucial downstream mediators, including NO and COX-2.

### 2.3. AVF and Its Active Constituents Inhibited the MAPK Signaling Pathway

**Figure 3:**
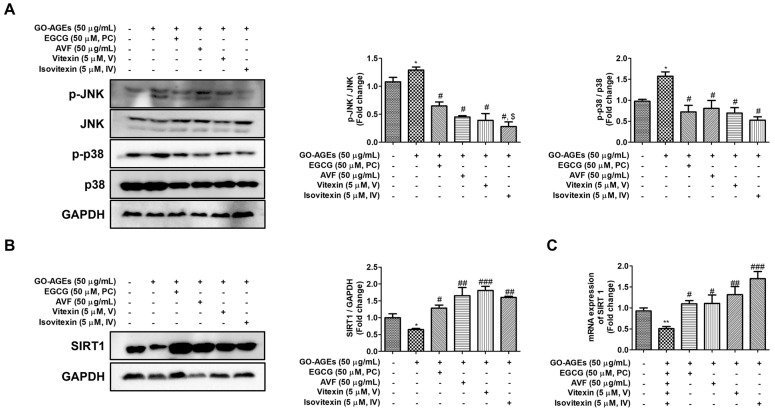
AVF and its active constituents modulate the MAPK and SIRT1 pathways in GO-AGE-induced HaCaT cells. (**A**) Representative Western blot images and densitometric quantification of the phosphorylation ratios of JNK (p-JNK/JNK) and p38 (p-p38/p38). (**B**) Representative Western blot images and densitometric quantification of the SIRT1 protein levels, normalized to those of GAPDH. (**C**) Relative mRNA expression of SIRT1 measured by qRT-PCR. Cells were treated with GO-AGEs (50 µg/mL) in the presence or absence of AVF (50 µg/mL), positive control (PC, EGCG 50 µM), vitexin (V 5 µM), or isovitexin (IV, 5 µM). Data are expressed as the mean ± SEM of three independent experiments (*n* = 3). * *p* < 0.05 and ** *p* < 0.01 vs. control; # *p* < 0.05, ## *p* < 0.01, and ### *p* < 0.001 vs. GO-AGE group; $ *p* < 0.05 vs. PC group.

In addition to the NF-κB pathway, the MAPK signaling cascade is another critical pathway that regulates inflammatory responses in keratinocytes. In this study, we focused on JNK and p38 as they are the key stress-activated protein kinases (SAPKs) specifically known to be triggered by oxidative stress and AGEs, whereas ERK is often associated with cell proliferation. We evaluated the phosphorylation of two key MAPK subfamilies as JNK and p38 where phosphorylation is associated with their activation. Western blot analysis revealed that stimulation with GO-AGEs significantly increased the phosphorylation of both JNK and p38 relative to their total protein levels (Figure 3A), confirming the activation of this pathway. Subsequent treatment with AVF, V, or IV markedly inhibited the phosphorylation of JNK and p38 (e.g., IV reduced p-JNK levels by approximately 50% compared to the GO-AGE group), indicating that these compounds blocked the upstream signaling cascade. Notably, treatment with V was particularly effective, causing p-JNK/JNK dephosphorylation to a comparable extent to the positive control group, despite the lower dosage used (5 µM vs. 50 µM). These findings highlight the ability of AVF and its constituents to modulate the MAPK pathway as part of their overall anti-inflammatory activity in skin cells.

### 2.4. AVF and Its Active Constituents Upregulated SIRT1 Expression

To investigate the role of sirtuins in the observed anti-inflammatory effects, we evaluated the expression of nuclear sirtuins (SIRT1, 2, 6, and 7) by qRT-PCR and Western blotting. While no significant changes were observed in the mRNA levels of SIRT2, SIRT6, or SIRT7, the expression of SIRT1 was significantly altered (Figure 3B and Figure S1). As shown in Figure 3B,C, treatment with GO-AGEs downregulated SIRT1 expression at both the mRNA and protein levels, which was reversed by subsequent treatment with AVF, V, or IV, all of which upregulated SIRT1 expression. Notably, treatment with V and IV induced particularly potent activation of SIRT1; specifically, IV upregulated SIRT1 protein expression by approximately 1.5-fold compared to the GO-AGE group, with expression levels significantly higher than those in both the GO-AGE and positive control groups.

### 2.5. Molecular Docking Analysis Confirmed Binding Affinities to Inflammatory Target Proteins

**Figure 4:**
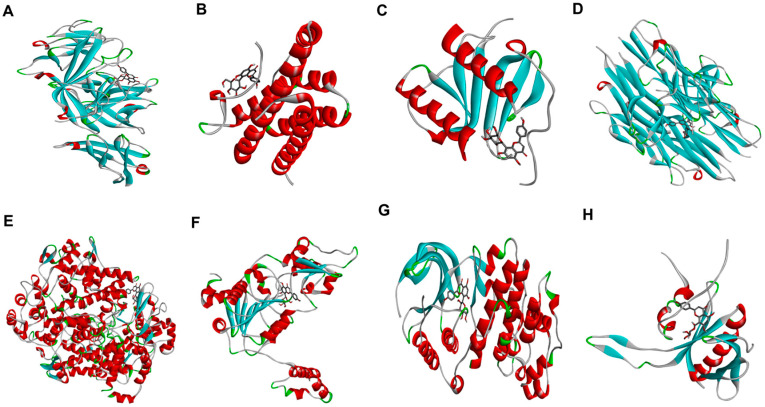
Molecular docking analysis of vitexin with key inflammatory target proteins. The figure shows the predicted binding conformations of vitexin with (**A**) IL-1β, (**B**) IL-6, (**C**) IL-8, (**D**) TNF-α, (**E**) COX-2, (**F**) SIRT1, (**G**) p38, and (**H**) p65. Target proteins are visualized as ribbons, and the ligand (vitexin) is visualized as sticks.

**Figure 5:**
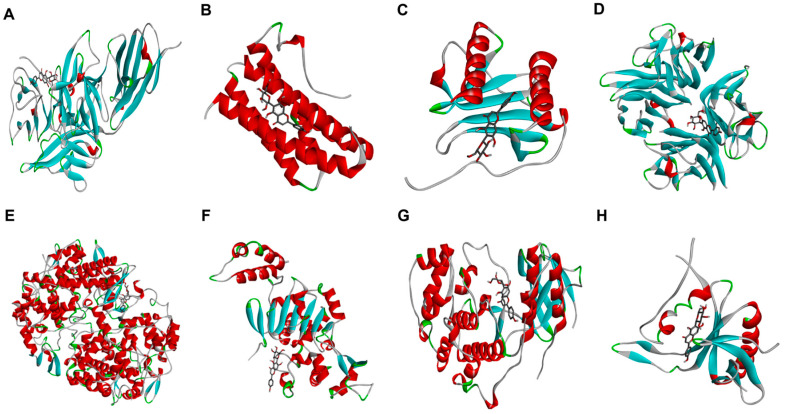
Molecular docking analysis of isovitexin with key inflammatory target proteins. The figure shows the predicted binding conformations of isovitexin with (**A**) IL-1β, (**B**) IL-6, (**C**) IL-8, (**D**) TNF-α, (**E**) COX-2, (**F**) SIRT1, (**G**) p38, and (**H**) p65. Target proteins are visualized as ribbons, and the ligand (isovitexin) is visualized as sticks.

To further understand the molecular interactions, a docking study was performed to evaluate the binding of the active phytoconstituents of the *Aloe* flower to key inflammatory target proteins. The results confirmed strong binding affinities for V and IV (Table 1, Figure 4, Figure 5, Figures S2 and S3). V exhibited strong binding affinities for all target proteins, including IL-1β (−7.8 kcal/mol), TNF-α (−8.5 kcal/mol), COX-2 (−8.9 kcal/mol), and p38 (−7.9 kcal/mol), with the highest binding affinity observed with SIRT1 (−9.4 kcal/mol), forming hydrogen bonds with key residues such as PHE (A:273), LYS (A:444), and ARG (A:274). IV also showed potent binding scores for IL-1β (−8.7 kcal/mol), TNF-α (−8.6 kcal/mol), p38 (−8.2 kcal/mol), and p65 (−8.2 kcal/mol). It displayed a particularly high binding affinity for COX-2 (−11.0 kcal/mol), with multiple hydrogen bonds stabilizing the interaction. The binding scores for IL-6 and IL-8 were lower for both compounds; they still suggested significant interaction capability. Thus, both V and IV demonstrated significant interactions with all target proteins, providing a structural basis for their ability to modulate signal transduction during skin inflammation. To provide deeper structural insights, the 2D interaction diagrams (Figures S2 and S3) were analyzed to identify key binding residues stabilized by hydrogen bonds. As shown in Figure S2, Vitexin interacts with IL-1β through hydrogen bonds with residues LYS A:27 and GLU B:10, while its interaction with COX-2 involves hydrogen bonding with CYS A:47 and SER A:49. Regarding Isovitexin (Figure S3), its high affinity for COX-2 (−11.0 kcal/mol) is supported by a dense network of hydrogen bonds involving ASN A:71, ASP A:133, and GLY A:135. Furthermore, the interaction between Isovitexin and SIRT1 is stabilized by hydrogen bonds with key residues such as LYS A:70 and ASP A:71. These specific molecular interactions provide a structural basis for the observed biological activities.

## 3. Discussion

AGEs are key pathological mediators that contribute to chronic inflammation and oxidative stress, particularly in the context of skin inflammation [31,32]. Dicarbonyl compounds, such as GO, which are precursors of GO-AGEs, play a crucial role in this process. Several in vitro studies have demonstrated the inflammatory effects of AGEs on skin cells such as keratinocytes and dermal fibroblasts by activating the NF-κB, MAPK, and RAGE signaling pathways. However, few studies have specifically investigated the effects of GO-AGEs [33,34]. In this study, we systematically investigated the protective, anti-inflammatory effects of AVF and its active constituents in a GO-AGE-induced skin inflammaging model.

Our findings demonstrate that the ethanol extract of AVF, along with its active flavonoids, V and IV, potently counteracted the inflammatory cascade initiated by GO-AGEs in HaCaT keratinocytes. This is consistent with previous reports highlighting the rich polyphenolic and flavonoid content of *Aloe vera* and its associated anti-inflammatory and antioxidant activities. Various phytoconstituents, including those from *Aloe vera* and *Brassica* species, inhibit protein glycation and act as carbonyl scavengers, making them promising therapeutic agents against AGE-related chronic illnesses [30,35,36,37]. Further, *Aloe vera* flower contains a significant quantity of isoflavones, including IO, V, and IV, which has been verified in several studies and detected by high-performance liquid chromatography (HPLC) [38,39,40]. In this study, we selected V and IV as active marker compounds based on these previously established quantitative profiles [39]. While we did not perform a specific quantitative analysis for the current extract batch, the differential effects observed between the crude extract (AVF) and the isolated compounds (e.g., in COX-2 suppression) suggest that other unquantified constituents, such as polysaccharides or anthraquinones, may interact antagonistically or synergistically within the complex matrix. Our results provide novel mechanistic insights into the anti-inflammatory effects of AVF and its active constituents (V and IV). Treatment with AVF, V, and IV inhibited the production of inflammatory cytokines (IL-6 and IL-8), NO, and COX-2, and suppressed MAPK signaling pathway activation and NF-κB expression, in addition to upregulating SIRT1 expression [35,41,42].

The release of pro-inflammatory cytokines is a hallmark of skin inflammation and plays a crucial role in the pathogenesis of various skin disorders, including psoriasis and erythema. In keratinocytes, stimuli such as AGEs trigger the production of key mediators including IL-6 as a significant factor in tissue injury and IL-8, whose release is often facilitated by IL-1β and TNF-α [43,44,45]. Our findings demonstrate that AVF and its active constituents effectively attenuated GO-AGE-induced IL-6 and IL-8 production at non-toxic concentrations (Figure 1 and Figure 2), validating the potent anti-inflammatory activity of *A. vera*. flowers in skin inflammation. Furthermore, nitric oxide synthetase (NOS) and COX-2 produce the inflammatory mediators NO and prostaglandins, respectively, and their overexpression is linked to chronic inflammatory diseases and cancer, making their suppression a key strategy for potential anti-inflammatory agents [31,46,47]. AVF and its active constituents effectively inhibited GO-AGE-induced NO production in HaCaT cells. Interestingly, while V and IV significantly inhibited COX-2 expression, the crude AVF extract did not (Figure 2). This discrepancy may be attributed to the complex composition of the crude extract. It is possible that other constituents within AVF exert antagonistic effects on the COX-2 pathway, or that the concentration of V and IV within the tested dose of AVF was insufficient to trigger COX-2 suppression, unlike the purified compounds.

The NF-κB pathway is a pro-inflammatory cascade that, upon activation, translocates NF-κB to the nucleus to initiate the transcription of numerous cytokines and chemokines [48]. Our results demonstrated that GO-AGEs potently activated this pathway in HaCaT cells, which was significantly attenuated by AVF, V, or IV treatment (Figure 2B,C). Similarly, the MAPK signaling pathway, that includes the key stress-activated kinases JNK and p38, is critical for inflammatory responses in keratinocytes [49,50,51]. Consistent with their effect on NF-κB, AVF, V, and IV significantly inhibited GO-AGE-induced phosphorylation of JNK and p38 (Figure 3A). Concurrent inhibition of these two major inflammatory signaling pathways provides a robust mechanistic explanation for the observed suppression of downstream cytokines and inflammatory mediators.

Further, we evaluated the expression of SIRT1 as a critical regulator of inflammatory responses and found that while other nuclear sirtuins were unaltered, SIRT1 expression was significantly increased at both the mRNA and protein levels following treatment with AVF, V, and IV (Figure 3B,C), suggesting that AVF and its active constituents upregulated SIRT1 expression [52]. Given that SIRT is known to inhibit NF-κB transcriptional activity, this upregulation is likely associated with the observed anti-inflammatory effects [20]. This finding is supported by the established mechanisms underlying SIRT1’s anti-inflammatory effects that involve direct deacetylation of the RelA/p65 subunit of NF-κB at lysine 310, which inhibits its transcriptional activity, thereby suppressing the expression of a wide array of pro-inflammatory genes, including cytokines and chemokines [20]. Therefore, by enhancing SIRT1 levels, AVF and its constituents engage a master regulatory pathway that governs cellular stress responses, providing a robust mechanistic basis for their anti-inflammatory effects and supporting their potential as therapeutic agents for inflammation in which SIRT1 dysfunction is often implicated [53].

EGCG, a major polyphenolic component of green tea with documented antioxidant and anti-inflammatory effects, was used as a positive control in this study [54]. Our results showed that AVF and its active constituents suppressed the production of pro-inflammatory cytokines (IL-6, TNF-α) and nitric oxide (NO), similar to the EGCG treatment group (Figure 1 and Figure 2). Notably, the active constituents demonstrated significant efficacy even at a 10-fold lower concentration (5 µM) compared to the positive control EGCG (50 µM). Specifically, treatment with V and IV decreased COX-2 expression and p-JNK/JNK phosphorylation to a level comparable to or exceeding that of the EGCG group. Furthermore, treatment with IV upregulated SIRT1 expression by 1.5-fold, suggesting distinct potential for modulating specific signaling pathways. Furthermore, treatment with IV upregulated SIRT1 expression by 1.5-fold, considerably more than that observed with the positive control under GO-AGE-induced inflammatory conditions.

**Figure 6:**
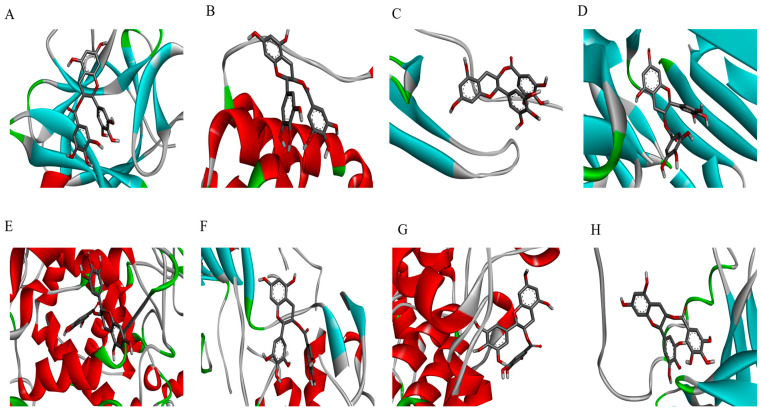
Molecular docking analysis of EGCG with key inflammatory target proteins. The figure shows the predicted binding conformations of EGCG with (**A**) IL-1β, (**B**) IL-6, (**C**) IL-8, (**D**) TNF-α, (**E**) COX-2, (**F**) SIRT1, (**G**) p38, and (**H**) p65. Target proteins are visualized as ribbons, and the ligand (EGCGw) is visualized as sticks.

In vitro molecular docking is a valuable tool for exploring the mechanisms underlying biological processes. Our docking results supported our experimental findings, showing that both V and IV have strong binding affinities for key inflammatory target proteins (Table 1, Figure 4, Figure 5, Figure 6, Figures S2 and S3). V exhibited the highest binding affinities towards SIRT1 (−9.4 kcal/mol) and COX-2 (−8.9 kcal/mol), and IV showed particularly strong binding to COX-2 (−11.0 kcal/mol) and IL-1β (−8.7 kcal/mol) in a comparision to EGCG (−7.7 and −10.8 kcal/mol respectively). On the other hand, EGCG has shown highest binding affinity towards COX-2 (−10.8 kcal/mol), and TNF-α (−8.8 kcal/mol) respectively.

For both compounds, binding was stabilized by several chemical bonds at multiple positions. The predicted binding affinities of both V and IV for these target proteins provide a structural basis for their ability to modulate the inflammatory signaling pathways involved in skin inflammation. Particularly, the exceptionally high binding affinity of IV for COX-2 (−11.0 kcal/mol) aligns with our in vitro findings, where IV demonstrated the most potent inhibition of COX-2 protein expression among the tested compounds (Figure 2). This consistency between in silico predictions and biological data reinforces the mechanism by which these flavonoids suppress inflammation.

Overall, our findings indicate that *A. vera* flower ethanol extract has significant potential as a therapeutic agent against GO-AGE-induced skin inflammaging. This effect is mediated by the inhibition of pro-inflammatory mediators via the MAPK and NF-κB signaling pathways. This study has several limitations that open avenues for future research. Our experiments were conducted using an in vitro model, in vivo studies are necessary to confirm the anti-inflammatory effects in a complex biological system. Future studies could use disease models, such as pruritus or dermatitis, in which exogenous glyoxal is involved. Furthermore, because skin damage is also caused by other compounds, such as advanced lipoxidation end products (ALEs), the effects of the extract against ALEs should also be investigated. Finally, the anti-glycation effects of *A. vera* extract and its active constituents should be evaluated under diabetic conditions, such as diabetic foot ulcers, where dicarbonyl reactive compounds are highly prevalent.

This study has several limitations that open avenues for future research. First, the experiments were conducted solely using an in vitro HaCaT model. While this immortalized cell line is a standard tool for screening keratinocyte biology, it may not fully replicate the complex environment of primary cells or in vivo skin tissue. Future research should validate these findings in animal models of skin inflammation or using primary keratinocytes. Second, we assessed signaling pathways at representative peak time points based on prior optimization, rather than providing a comprehensive time-course analysis. Third, while we identified active constituents based on prior profiling, a specific quantitative analysis of the current extract batch was not performed. Additionally, while we focused on the downstream MAPK and NF-κB pathways, the direct involvement of the RAGE receptor, a known target of GO-AGEs [14,16], was not measured in this study. Future research should confirm the direct impact of AVF on RAGE expression and validate the SIRT1-NF-κB axis using loss-of-function approaches. Finally, future studies could broaden the scope by investigating the effects against other glycation products such as ALEs or evaluating efficacy under diabetic conditions.

## 4. Materials and Methods

### 4.1. Chemicals and Reagents

Dried *Aloe vera* flowers (UV-AVF1001) were provided by Univera Co., Ltd. (Seoul, Republic of Korea). Dulbecco’s modified Eagle’s medium (DMEM), fetal bovine serum (FBS), and penicillin-streptomycin (P/S) were purchased from Gibco (Grand Island, NY, USA). Glyoxal solution (GO), sodium azide, and 3-(4,5-dimethylthiazol-2-yl)-2,5-diphenyltetrazolium bromide (MTT) were purchased from Sigma-Aldrich (St. Louis, MO, USA). The enzyme-linked immunosorbent assay (ELISA) kits for IL-6 and TNF-α were obtained from R&D Systems Inc. (Minneapolis, MN, USA). PRO-PREP™ protein extraction solution and the enhanced chemiluminescence (ECL) detection kit were from Intron Biotechnology (Seongnam, Republic of Korea). Primary antibodies against glyceraldehyde 3-phosphate dehydrogenase (GAPDH), COX-2, p38, phospho-p38 (p-p38), JNK, phospho-JNK (p-JNK), kappa-light-chain-enhancer of activated B cells (NF-κB), SIRT1, along with horseradish peroxidase-conjugated secondary antibodies were purchased from Santa Cruz Biotechnology (Dallas, TX, USA), Cell Science (Canton, MA, USA), and Cell Signaling Technology (Beverly, MA, USA).

### 4.2. Extract Preparation

Dried *A. vera* flowers (AVF, 1 g) were powdered and extracted using absolute ethanol. The mixture was sonicated at 40 °C for 60 min, filtered, and centrifuged again. The solvent was removed using a rotary evaporator, and the resulting extract was freeze-dried. The final extract was stored at −4 °C until use. AVF yield was 34.01%.

### 4.3. Cell Culture, Maintenance, and Treatment

HaCaT cells were attained from the Korean Cell Line Bank (Seoul, Republic of Korea) and cultured in high-glucose DMEM supplemented with 10% heat-inactivated FBS and 1% P/S. The cultured cells were maintained at 37 °C and 5% CO_2_ in a humidified incubator [28].

For the treatment of cells, AVF was first dissolved in phosphate-buffered saline (PBS, pH 7.4) and filtered to make stock solution of 10 mg/mL. Then, it was further diluted to 50 μg/mL, and the active constituents (IO, V, and IV) of AVF were diluted to 5 μM in serum-free DMEM media for cell treatment. Epigallocatechin gallate (EGCG, 50 μM), a well-known anti-inflammatory agent was used as a positive control in this study [55]. GO-AGEs were prepared by incubating bovine serum albumin (5 mg/mL) and 0.2% sodium azide with 10 mM Glyoxal in PBS (pH 7.4), at 37 °C for 15 days in the absence of light. After incubation, the solution was dialyzed, filtered through desalting columns, lyophilized, and stored until further use. Then, the stock solution of GO-AGEs (10 mg/mL) was prepared in serum-free media and further diluted to 50 µg/mL before the treatment. In our previous studies utilizing this protocol, we confirmed that native BSA (without glyoxal reaction) did not induce significant cytotoxicity or inflammatory responses compared to vehicle control [14,16]. Therefore, GO-AGEs were used as the primary inflammatory stimulus in this study.

### 4.4. Cell Viability Assay

HaCaT cells were cultured in 96-well plates (4 × 10^4^ cells/well) with further incubation was done for 24 h. Then, the cells were treated with different concentrations of AVF, V, and IV for 24 h. After that, the media was replaced with 0.5 mg/mL MTT solution, with further incubation for 1 h at 37 °C. Then, the MTT solution was removed, with immediate addition of 100 μL, DMSO to each well. The absorbance was measured by using a microplate reader at 570 nm (Molecular Devices E09090; Molecular Devices, San Francisco, CA, USA).

### 4.5. ELISA for the Detection of IL-6 and TNF-α

For the detection of IL-6 and TNF-α by ELISA, HaCaT cells were seeded into 96-well plates (3 × 10^4^ cells/well) and co-treated with GO-AGEs (50 µg/mL) and the samples (AVF, V, IV) subsequently for 24 h. Then, the secreted IL-6 and TNF-α in supernatants were measured by using ELISA kits according to the manufacturer’s instructions.

### 4.6. Determination of Nitric Oxide (NO) Production

HaCaT cells (3 × 10^4^ cells/well) were cultured for 24 h and then treated with GO-AGEs (50 µg/mL) followed by AVF, V, IV for 24 h additionally. The secretion of nitrite in the culture medium is considered as an indicator of NO production, which was measured by using Griess reagent (1% sulfanilamide and 0.1% naphthyl ethylenediamine dihydrochloride in 2.5% phosphoric acid) following previously described method [16,55]. The absorbance of nitrite was checked by using microplate reader (Molecular Devices E09090; Molecular Devices, San Francisco, CA, USA) at 540 nm.

### 4.7. Western Blotting

HaCaT cells were subjected towards harvesting and then washing were done with cold PBS (pH 7.4), with further lysis by PRO-PREP lysis buffer (containing protease and phosphatase inhibitors). Then, the protein concentration was determined via the Bradford assay [45]. Briefly 30 µg of protein from each group were separated by 6–10% sodium dodecyl sulfate polyacrylamide gel electrophoresis (SDS-PAGE) and then, transferred to a nitrocellulose membrane. The membrane was blocked with 5% skim milk and incubated with primary antibodies (against GAPDH, COX-2, SIRT1, NF-κB, p-p38, p38, p-JNK, and JNK) overnight at 4 °C. Then, the incubation was done with horseradish peroxidase-conjugated secondary antibodies, and the protein bands were visualized by using an enhanced chemiluminescence reagent in ChemiDoc XRS+ imaging system (Bio-Rad, Hercules, CA, USA). Finally, the densitometric analysis was performed by Image Master^TM^ 2D Elite software version 3.1 (Amersham Pharmacia Biotech, Piscataway, NJ, USA).

### 4.8. Quantitative Real-Time PCR (qRT-PCR)

Total RNA was extracted from HaCaT cells (6-well plates, 3 × 10^5^ cells/well) by using an RNA extraction kit (KeyGEN Biotech, Nanjing, China). The cDNA synthesis was done from total RNA and then, qRT-PCR was performed by using the PrimeScript RT Reagent Kit (Takara, Kusatsu, Shiga, Japan) following the manufacturer’s instructions [46]. The following primer sequences were used for qRT-PCR are given hereby: IL-6 (forward: 5′-AACCTGAACCTTCCAAAGATGG-3′, reverse, 5′-TCTGGCTTGTTCCTCACTACT-3′); IL-8 (forward: 5′-CATACTCCAAACCTTTCCACCCC-3′, reverse, 5′-TCAGCCCTCTTCAAAAACTTCTCCA-3′); SIRT1 (forward: 5′-CTATACCCAGAACATAGACACG-3′, reverse, 5′-ACAAATCAGGCAAGATGC-3′); SIRT2 (forward: 5′-CCATCTGTCACTACTTCATGC-3′, reverse, 5′-AAGTCCTCCTGTTCCAGC-3′; SIRT 6, forward: 5′-AGGGACAAACTGGCAGAGC-3′ reverse, 5′-TTAGCCACGGTGCAGAGC-3′); SIRT 7, (forward: 5′-GCAGAGCAGACACCATCC-3′, reverse, 5′-GTTCACGATGTAAAGCTTCG-3′); GAPDH (forward: 5′-TCCACTGGCGTCTTCACC-3′, reverse: 5′-GGCAGAGATGATGACCCTTTT-3′). The expression levels of mRNA of IL-6, IL-8, and SIRT1,2,6,7 were normalized to GAPDH.

### 4.9. In Vitro Molecular Docking Analysis

To predict the binding confirmation of ligands, in vitro molecular docking analysis were performed by the AutoDock Vina software program (version 1.1.2) as previously reported, The 2D structures of active constituents of AVF, V (PubChem CID: 5280441), IV (PubChem CID: 162350) and EGCG (PubChem CID: 65064) were attained from the PubChem database (https://pubchem.ncbi.nlm.nih.gov/, accessed on 3 February 2023) [16,37]. Then, the 3D structures of different target proteins were obtained from the Protein Data Bank (PDB) (https://pubchem.ncbi.nlm.nih.gov/, accessed on 3 February 2023), including IL-1β (PDB ID:1ITB), IL-6 (PDB ID:1ALU), IL-8 (PDB ID:1IL8), TNF-α (PDB ID:2AZ5), COX-2 (PDB ID:5KIR), SIRT1 (PDB ID:4ZZJ), P38(PDB ID: 1A9U), and P65 (PDB ID:5URN). Initially, the proteins and ligands were prepared in PDB format, partial charge, and atom-type (PDBQT) formats, and finally docking was performed using the Lamarckian Genetic Algorithm. Then, the protein–ligand complexes having the lowest binding energies were selected and visualized by using Discovery Studio Visualizer (version 16.1.0.15350) to interpret the interactions.

### 4.10. Statistical Analysis

One-way analysis of variance (ANOVA) was performed using GraphPad Prism 5 (GraphPad Software Inc., La Jolla, CA, USA) to make comparison among various treatment groups, and *p*-value < 0.05 were accounted as statistically significant. The obtained results are presented as mean ± standard error of the mean (SEM) from three different independent experiments.

## 5. Conclusions

In conclusion, this study demonstrates that AVF and its active constituents (V and IV) possess potent anti-inflammatory properties in a GO-AGE-induced keratinocyte model. The underlying mechanism involves the suppression of pro-inflammatory cytokines (IL-6 and IL-8) and the inhibition of their associated MAPK and NF-κB signaling pathways. Furthermore, these compounds inhibit COX-2 expression and upregulated the anti-inflammatory protein SIRT1. These in vitro findings, supported by molecular docking analyses, provide a strong rationale for further in vivo investigation of AVF and its active constituents as effective agents for treating skin inflammaging and skin conditions.

## Figures and Tables

**Table 1:** Molecular docking scores of vitexin (V) and isovitexin (IV) against key inflammatory target proteins.

Target Proteins	Ligands	Docking Score (kcal/mol)	H-Bond
IL-1β (PDB ID:1ITB)	Vitexin	−7.8	LYS A:27; GLU B:10; MET A:20; GLY A:22; GLU B:11
	Isovitexin	−8.7	LYS A:77, LEU A:134, LYS A:77, LEU A:8; VAL A:132
	EGCG	−7.3	ALA A:135; ILE A:136; THR A:137; THR A:89; GLU A:93; ASP A:134; LEU A:133; THR A:82; LYS A:70; ASP A:71
IL-6 (PDB ID:1ALU)	Vitexin	−6.4	ARG A:182; GLN A:175
	Isovitexin	−6.8	GLU A:51; ASP A:71; LYS A:70
	EGCG	−6.3	SER A:125; MET A:130; PHE A:135; PRO A:131; LYS A:74; LYS A:77; LEU A:26; LEU A:80; LEU A:82; VAL A:72; VAL A:132; GLU A: 25; GLN A: 81; THR A:79; TYR A:24
IL-8 (PDB ID:1IL8)	Vitexin	−7.3	GLN B:59
	Isovitexin	−7.1	LEU A:51; ALA B:69; GLU B:70
	EGCG	−6.4	HIS A:33; LEU A:5; ARG A:6; THR A:12; LYS A:11; ILE A:10; CYS A:9; CYS A:7; PRO A:53; THR A:37; ASN A:36
TNF-α (PDB ID:2AZ5)	Vitexin	−8.5	ALA D:111; GLU D:116; SER C:99; PRO C:100; HIIS C:73
	Isovitexin	−8.6	SER C:60; LEU C:120; THR A:37
	EGCG	−8.8	GLN C:61; GLN D:61; PRO C:117; ILE C:58; ILE C:80; ILE C:118; THR C:79; TYR C:79; TYR D:151; ALA C:96; SER C:95; HIS C:78; VAL C:123
COX-2 (PDB ID:5KIR)	Vitexin	−8.9	CYS A:47; SER A:49
	Isovitexin	−11.0	GLU B:93; ASN A:71; ASP A:133; GLY A:135; CYS A:47
	EGCG	−10.8	ALA B:202; THR B:206; TYR B:385; GLN B:289; PHE B:210; LYS B:211; GLN B:203; HIS B:388; HIS B:386; HIS B:214; THR B:212; GLN B:454; ALA B:450; VAL B:447; SER B:451
SIRT1 (PDB ID:4ZZJ)	Vitexin	−9.4	PHE A:273; LYS A:444; ARG A:274
	Isovitexin	−7.1	LYS A:70; ASP A:71; GLU A:93
	EGCG	−7.7	GLN A:421; GLN 4:361; GLY A:364; GLU A:416; LEU A:418; LYS A:408; LYS A:375; LYS A:377; PHE A:413; ASN A:417; THR A:368; ALA A:367; PRO A:409; SER A:365; SER A:370
P38 (PDB ID: 1A9U)	Vitexin	−7.9	ALA A:172; ARG A:149; ARG A:173; TYR A:200; SER A:326
	Isovitexin	−8.2	ASP A:168; LYS A:53; PHE A:169; ARG A:67; ARG A:173; LEU A:104; LEU A:75; LEU A:86; VAL A:105; TYR A:35
	EGCG	−7.9	ILE A:297; LYS A:295; LYSA: 287; GLU A:286; PHE A:270; LYS A:267; LEU A:289; VAL A:290; VAL A:239; LEU A:246; GLU A: 245; GLY A:243; ASP A:292; PRO A:242; TRP A:207
P65 (PDB ID:5URN)	Vitexin	−7.4	HIS A:87; GLU A:10
	Isovitexin	−8.2	ASP B:531; LYS A:11
	EGCG	−7.9	GLY B:526; ASN B: 525; MET A:59; MET A:88; LEU A; 60; LYS A:11; PHE B:534; ASP B; 531; GLU B: 532; GLU A:10; SER A:90; PHE A:9; PHE A:9; ILE A:8; LEU A:60; PRO B:524

## Data Availability

The original contributions presented in this study are included in the article/Supplementary Materials. Further inquiries can be directed to the corresponding authors.

## Supplementary Materials

The following supporting information can be downloaded at https://www.mdpi.com/article/10.3390/ph19010121/s1, Figure S1: Effect of AVF and its active constituents on the expression of nuclear sirtuins (2, 3, and 7) in GO-AGE-induced HaCaT cells; Figure S2: Predicted binding interactions of vitexin (V) with target proteins. The figure shows the molecular docking results of V with (A) IL-1β, (B) IL-6, (C) IL-8, (D) TNF-α, (E) COX-2, (F) SIRT1, (G) p38, and (H) p65; Figure S3: Predicted binding interactions of isovitexin (IV) with target proteins. The figure shows the molecular docking results of IV with (A) IL-1β, (B) IL-6, (C) IL-8, (D) TNF-α, (E) COX-2, (F) SIRT1, (G) p38, and (H) p65. Supplemental material for this article is available online.
