# Supplementary material for: Exploring the Anti-Inflammatory Effects of Aloe vera Flower (AVF) and Its Active Ingredients in a Skin Inflammation Model Induced by Glyoxal-Derived Advanced Glycation End Products (GO-AGEs)
Source: Pharmaceuticals (Basel). 2026 Jan 9;19(1):121. doi: 10.3390/ph19010121 (PMC12845024; doi:10.3390/ph19010121)
Supplement: Supplementary file 1 [file pharmaceuticals-19-00121-s001.zip › pharmaceuticals-4093023-supplementary.pdf]

Supplementary materials

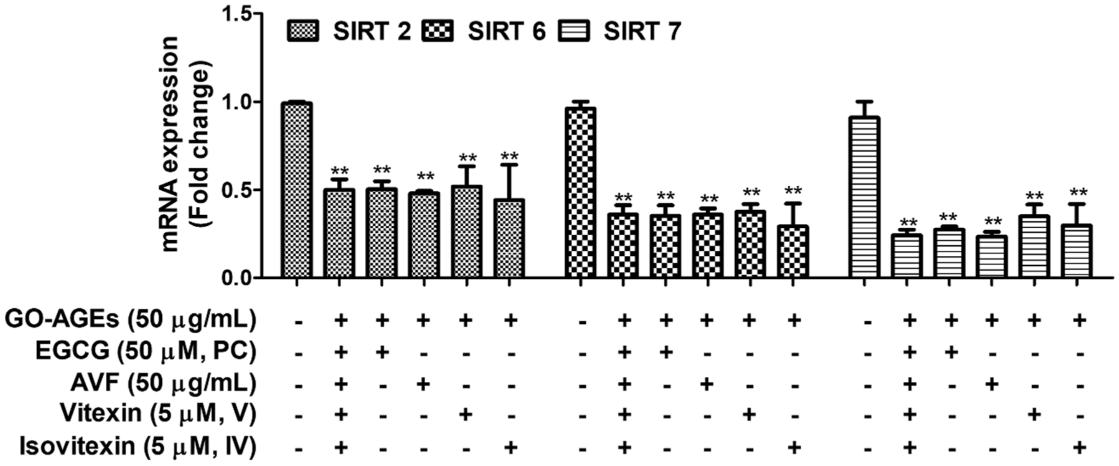

Figure S1. Effect of AVF and its active constituents on the expression of nuclear sirtuins (2, 3, and 7) in GO-AGEs-induced HaCaT cells. The relative expression levels of SIRT2, 3, and 7 were measured by qRT-PCR. Cells were treated with GO-AGEs (50 μg/mL) in the presence or absence of AVF (50 μg/mL), positive control (PC, EGCG 50 μM), vitexin (V 5 μM), or isovitexin (IV, 5 μM). Data are expressed as the mean ± SEM of three independent experiments (n=3). $^{*}p < 0.05$ and $^{**}p < 0.01$ vs. control; $^{\#}p < 0.05$, $^{\#\#}p < 0.01$, and $^{\#\#\#}p < 0.001$ vs. GO-AGEs group; $^{\$}p < 0.05$ vs. PC group.

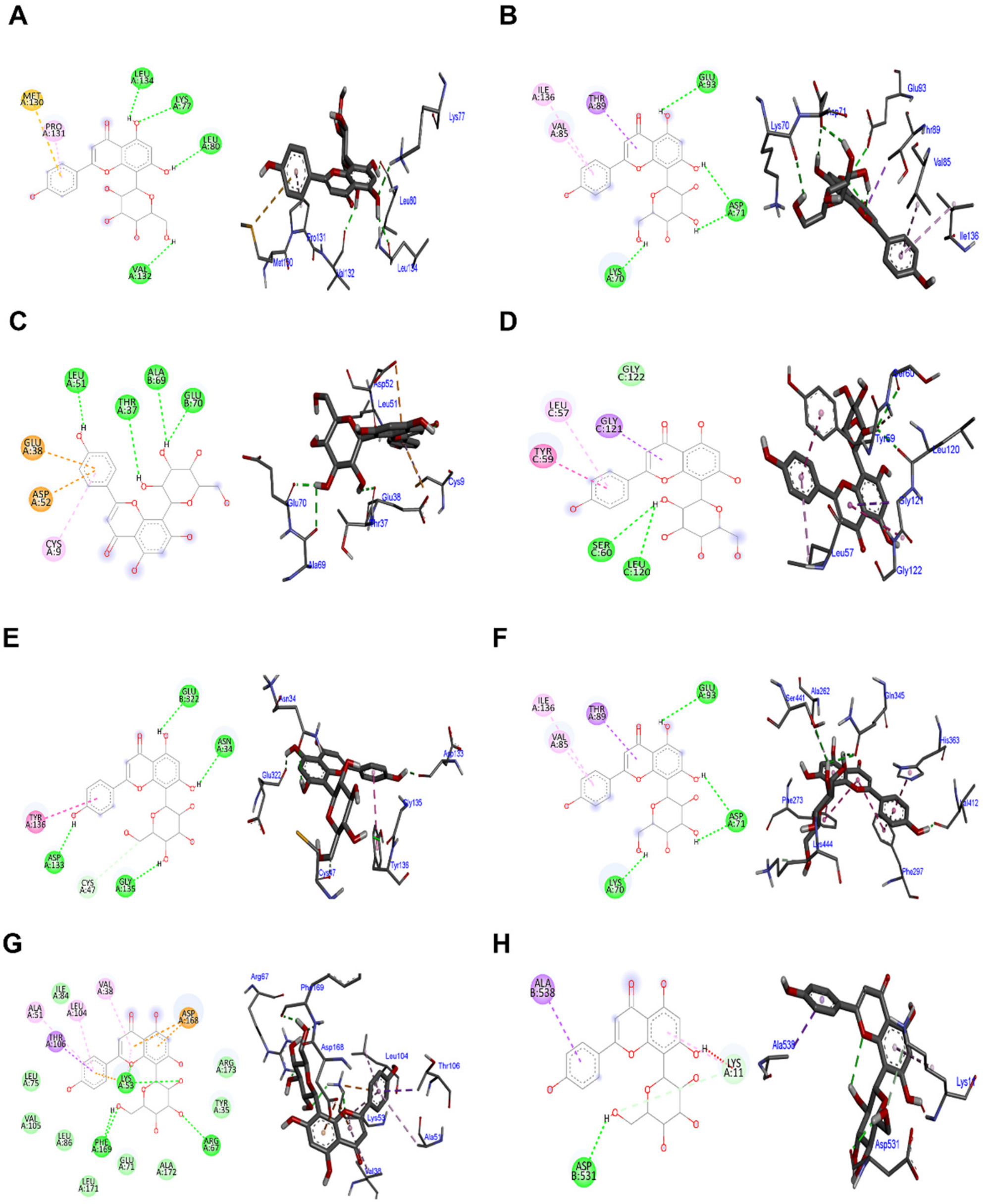

Figure S2. Predicted binding interactions of vitexin (V) with target proteins. The figure shows the molecular docking results of V with (A) IL-1β, (B) IL-6, (C) IL-8, (D) TNF-α, (E) COX-2, (F) SIRT1, (G) p38, and (H) p65. The left panels display the pocket binding view, and the right panels illustrate the detailed binding interactions, including different types of bonds observed during complex formation.

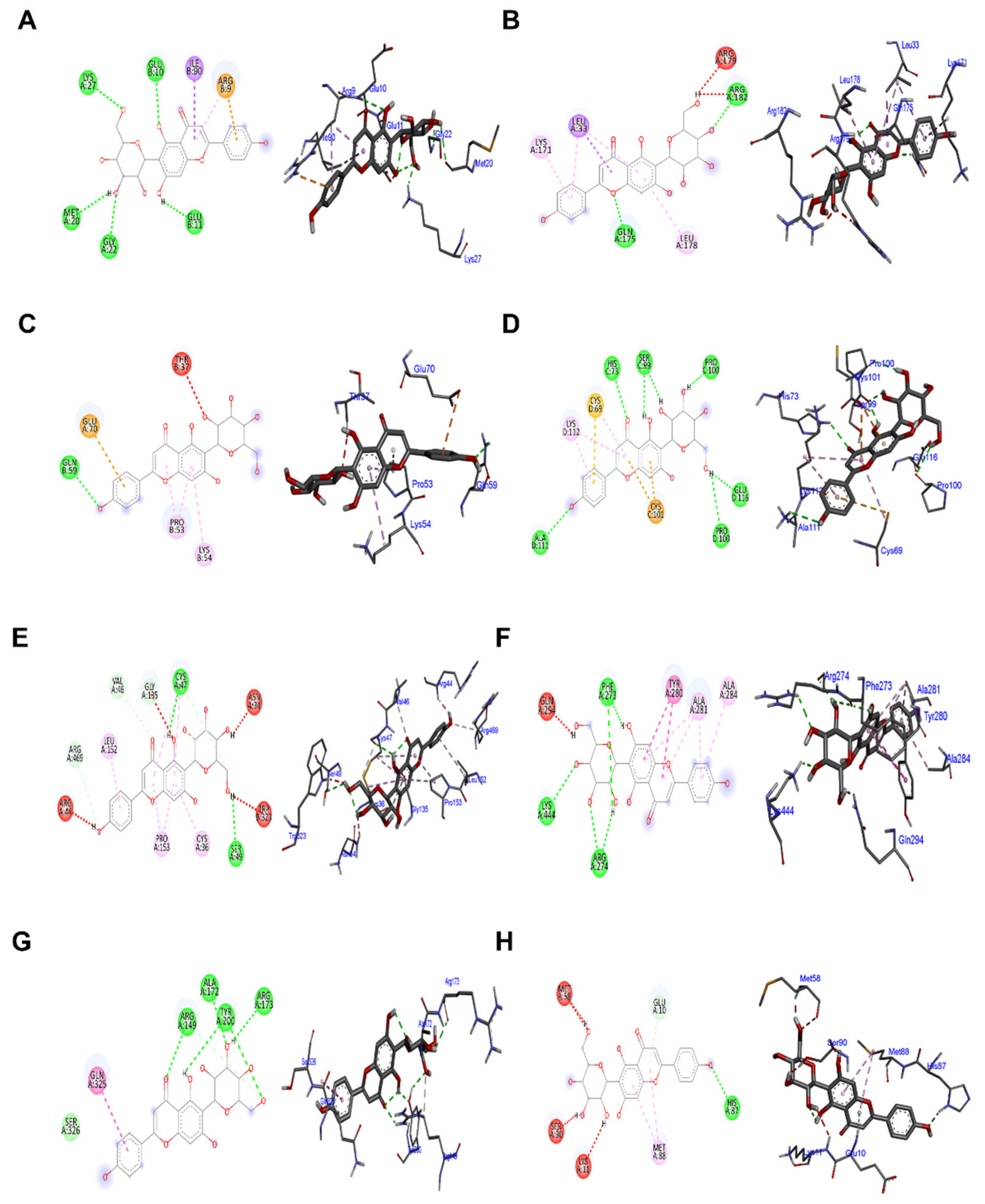

Figure S3. Predicted binding interactions of isovitexin (IV) with target proteins. The figure shows the molecular docking results of IV with (A) IL-1β, (B) IL-6, (C) IL-8, (D) TNF-α, (E) COX-2, (F) SIRT1, (G) p38, and (H) p65. The left panels display the pocket binding view, and the right panels illustrate the detailed binding interactions, including different types of bonds observed during complex formation.
